# Supplementary material for: Innovative Collaboration between a Medical Clinic and a Community Pharmacy: A Case Report
Source: Pharmacy (Basel). 2019 Jun 14;7(2):62. doi: 10.3390/pharmacy7020062 (PMC6630453; doi:10.3390/pharmacy7020062)
Supplement: Supplementary file 1 [file pharmacy-07-00062-s001.pdf]

Table S1. Medication-Related Problems Identified during CMRs (N = 116).

Medication-Related Problem (MRP)	Frequency (%)
Needs additional therapy	45 (38.8)
Suboptimal therapy	22 (19.0)
Unnecessary therapy	18 (15.5)
Nonadherence	14 (12.1)
Dose too low	13 (11.2)
Adverse drug reaction	10 (8.6)
Inappropriate administration/technique	8 (6.9)

Table S2. Pharmacist Actions For Medication-Related Problems (N = 116).

Pharmacist Action	Frequency (%)
Change drug	21 (18.1)
Initiated new therapy	16 (13.8)
Discontinued therapy	15 (12.9)
Altered administration/technique	13 (11.2)
Increased dose	9 (7.7)
